# Prediction and experimental validation approach to improve performance of novel hybrid bio-inspired 3D printed lattice structures using artificial neural networks

**DOI:** 10.1038/s41598-023-33935-0

**Published:** 2023-05-12

**Authors:** Ramakrishna Doodi, Bala Murali Gunji

**Affiliations:** School of Mechanical Engineering, Vellore Institute of Technology, Vellore, 632014 Tamil Nadu India

**Keywords:** Mathematics and computing, Engineering, Mechanical engineering

## Abstract

Novel Cellular lattice structures with lightweight designs are gaining more interest in the automobile and aerospace sectors. Additive manufacturing technologies have focused on designing and manufacturing cellular structures in recent years, increasing the versatility of these structures because of the significant benefits like high strength-to-weight ratio. In this research, a novel hybrid type of cellular lattice structure is designed, bio-inspired from the circular patterns seen in the bamboo tree structure and the overlapping patterns found on the dermal layers of fish-like species. The unit lattice cell with varied overlapping areas with a unit cell wall thickness of 0.4 to 0.6 mm. Fusion 360 software models the lattice structures with a constant volume of 40 × 40 × 40 mm. Utilizing the stereolithography (SLA) process and a vat polymerization type three-dimensional printing equipment is used to fabricate the 3D printed specimens. A quasi-static compression test was carried out on all 3D printed specimens, and the energy absorption capacity of each structure was calculated. Machine learning technique like the Artificial neural network (ANN) with Levenberg–Marquardt Algorithm (ANN-LM) was applied to the present research to predict the energy absorption of the lattice structure with parameters such as overlapping area, wall thickness, and size of the unit cell. The k-fold cross-validation technique was applied in the training phase to get the best training results. Overall, the results obtained using the ANN tool are validated and can be a favourable tool for lattice energy prediction with available data.

## Introduction

Bioinspiration involves creating unique structures and materials by utilising biological systems' knowledge and the evolution and development of biological systems throughout millions of years. Physical system modelling and simulation were enhanced to capture natural structural characteristics for application in cutting-edge bioinspired designs. On the other hand, bio-mimicry aims to replicate biological materials' designs, unlike bio-inspiration. The study of the particular abilities that distinguish some creatures from others is the goal of the discipline of bioinspired research, which pays homage to the classical foundations of science. Most scholars have used relevant real-world projects created using bioinspired designs to describe these techniques. A problem-driven, bio-inspired design approach necessitates using various technologies, some of which are difficult and need substantial training, while others are easy to use and understand^1^. Increasing the energy absorption capacity of lightweight constructions without compromising stiffness and strength has long been challenging since many potential uses might have a significant influence.

In Particular, performance, safety, cost, and environmental effect are significantly impacted in contexts with volume or weight restrictions. These problems are addressed by an emerging class of discrete open-cell lattices that provide superior advantages over solid materials, such as lightweight design, high permeability, and immediate command over regional architecture. This control may increase the structure's efficacy by optimising the material distribution inside the volume^2^. Dimensional accuracy, residual stresses, surface quality, and microstructures are some of the main benefits of lattice structures from a design standpoint. These factors influence the mechanical performance of the entire structure and other crucial characteristics like surface area, porosity, fluid dynamics, and material usage^3^. Therefore, adjusting these design characteristics is vital for optimizing and predicting how these structures behave in specific applications. Most earlier research^4–7^ concentrated on the connection between the mechanical characteristics of naturally occurring structures and their structural geometries, which commonly exhibit similar patterns. Examples of these structures include turtle shells, trabecular bone, horns, conch shells, balanus shells, fish scales, spider webs, plant stems, bamboo tree structures, wood, etc. Low-weight periodic open lattice structures made of femur bone and bamboo are widely employed in aerospace, automotive, and medical applications due to their enhanced performance^8,9^. These occurrences lead scientists to develop state-of-the-art geometries for cellular lattice structures to enhance energy absorption. The mechanical behaviour of the lattice structure can be influenced by the unit cell shape, cell size, relative density, and manufacturing method^10^. The open-type strut-based lattice structure can enhance mechanical qualities with controlled stiffness, more excellent SEA, and more extended plateau stresses than closed shell-based lattice structures^11^. Several publications^12,13^ summarise the current level of knowledge about the mechanical behaviour and structure of cells. Other publications^14–16^ provide examples of cellular structures' design, creation, and testing for uses. To successfully develop computer-aided design (CAD) models of lightweight truss structures and cellular unit cells and-optimize optimize the structures to have superior stiffness, strength, and weight attributes, further work is currently being done^17,18^. According to the studies mentioned in the research^12^, the topology or form of the cell, the properties of the material, and the lattice structure's relative density are the three key factors impacting the features of cellular structures. In addition, the above study^12^ aims to examine the compressive and flexural characteristics of various unit cell layouts. These qualities are experimentally determined using straightforward compressive, flexural (or bend), and fatigue testing. The major goal of all the literature discussed above is to understand the properties of unit cells well enough to quickly and effectively replace solid parts with lattice-like structures and develop novel components that entirely use lattice structures.

Designers, manufacturers, and several other entities are adopting additive manufacturing (AM), changing the business. A prototype should undergo several processes before commencing AM development since it may drastically cut industry lead times. Designers may navigate the many AM techniques and materials with the help of these procedures. The market has been more accessible to a variety of material portfolios, including plastics, metals, and ceramics, as a result of the introduction of many AM methods, including stereolithography (SLA), selective laser sintering (SLS), and fused deposition manufacturing (FDM)^19^. Numerous research investigating material anisotropy as a benefit for structural or mechanical performance have focused on customising features using additive manufacturing^20^. Most articles focus on the mechanical characteristics of FDM components, even though SLA is one of the most well-established AM techniques; this is likely because FDM was developed more quickly and at a lower cost. Internal cavities and a lack of surface precision are two limitations of FDM^21^. The literature^6^ also discusses and compares every additive manufacturing process relevant to creating cellular lattice structures. The fast growth of additive manufacturing) technology has opened the possibility of fabricating essential complex lattice-like structures with enhanced uniformity and resolution.

The VAT Photo polymerization technique is one of the existing additive manufacturing techniques that is reasonably quick, has a superior resolution, and has good surface polish^22^. The first vat photopolymerization approach was SLA, developed in the early 1980s. This AM technique creates the required type of entity layer by layer by selectively curing liquid-type photopolymers through a polymerization process triggered by light. Materials for most vat photopolymerization processes must adhere to two essential criteria: excellent fluidity and photo curability^23^. Photopolymers often have initiators that can respond to particular illumination. A comparison analysis has been made between nature-inspired 2.5-Dimensional nature-inspired cellular structure and nature-inspired novel flower lattice. It is attained superior specific energy absorption capacity^24,25^.

Artificial Neural Networks are a unique machine learning algorithm inspired by how the human brain functions. ANNs are a feature of the rapidly developing field of deep learning in machine learning. That is, the ANN can learn from the data in a way comparable to how the neurons in our nervous system can learn from past data and provide replies in the form of predictions or categorizations. ANNs aim to identify novel patterns by displaying a complicated connection between inputs and outputs. These artificial neural networks are used for various activities, including image identification, speech recognition, machine translation, and medical diagnosis. The ability of ANN to learn from example data sets is a crucial benefit. The most frequent application of ANN is a rough approximation of a random function. These kinds of technologies enable one to specify the distribution's solutions cost-effectively. Instead of using the complete dataset, ANN can also use a sample of the data to get the result. Due to ANNs' superior predictive powers, one may use them to improve already used data analysis approaches. An ideal neural network has three layers: (1) Input Layers—The input layer, the top layer of an ANN, is where input data is received initially. This data can take the shape of words, digits, audio files, picture frames, etc. (2) Hidden Layers: The ANN model's centre contains the hidden layers. It is also conceivable to have one hidden layer, like a perceptron, or several hidden layers. These hidden layers perform several mathematical operations on the incoming data to find any patterns. (3) Output Layer: We may obtain the desired result in the output layer by carefully computing the intermediate layer. To predict appropriate values, the activation function of neurons in the hidden and output layers is critical^26^. In this paper, ANN indicates the capability of energy absorption of the novel hybrid cellular lattice structures, which may be an initial approach in lightweight lattice designs.

## Methodology for design and fabrication of novel hybrid bio-inspired lattice structures (BILS)

**Figure 1:**
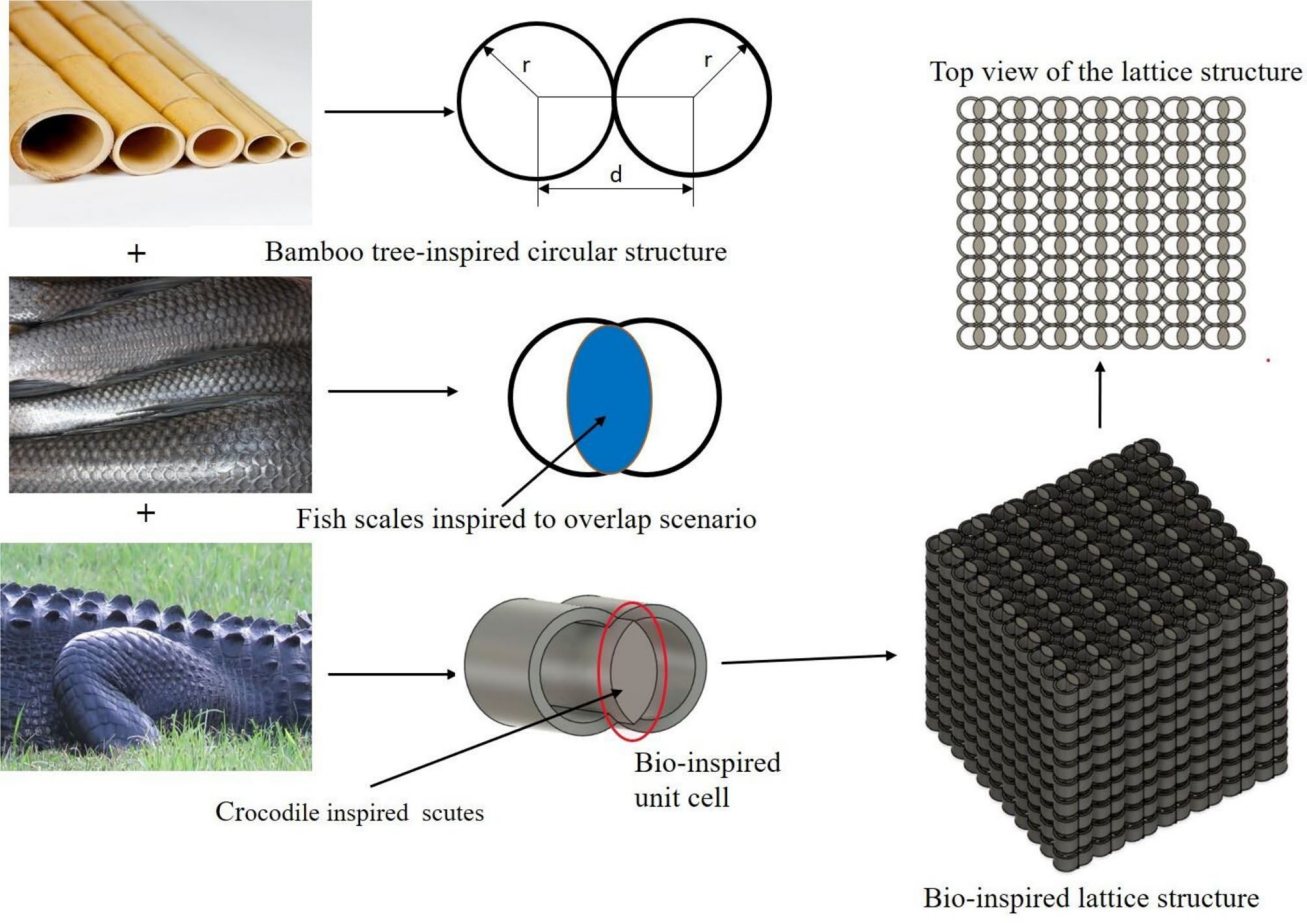
Evolution of Novel Hybrid Bio-inspired lattice cellular structure from the bamboo tree, Fish scales, and scutes observed from crocodile skin.

The superior mechanical strength observed on fish and other aquatic species scales is perfect for building lightweight structures. The literature^9,27–34^ uses straightforward design, production, and testing techniques related to the scales present in dermal layers of aquatic animals. The bamboo tree-like structure with high specific energy absorption (SEA) applications, as noted in the literature^10,35,36^, is another typical structure. Alligators and crocodiles have bump-like projecting structures called scutes on their dermal layers. These scutes are far more sensitive than human fingers and make great pressure detectors. A thorough examination of the scutes seen on the upper dermal layers of the many species of crocodiles, alligators, and turtle shells found on Earth's surface^9,37,38^. The scutes found on the upper dermal layers of some species of crocodilians, bamboo tree structures, and aquatic species' scales were all used as inspiration for constructing a unique hybrid kind of cellular lattice structure used in this study. The concept is shown in the Fig. 1 illustrates the idea behind structuring a hybrid novel lattice structure for energy absorption applications.

### Geometric modelling and tessellation methodology

**Figure 2:**
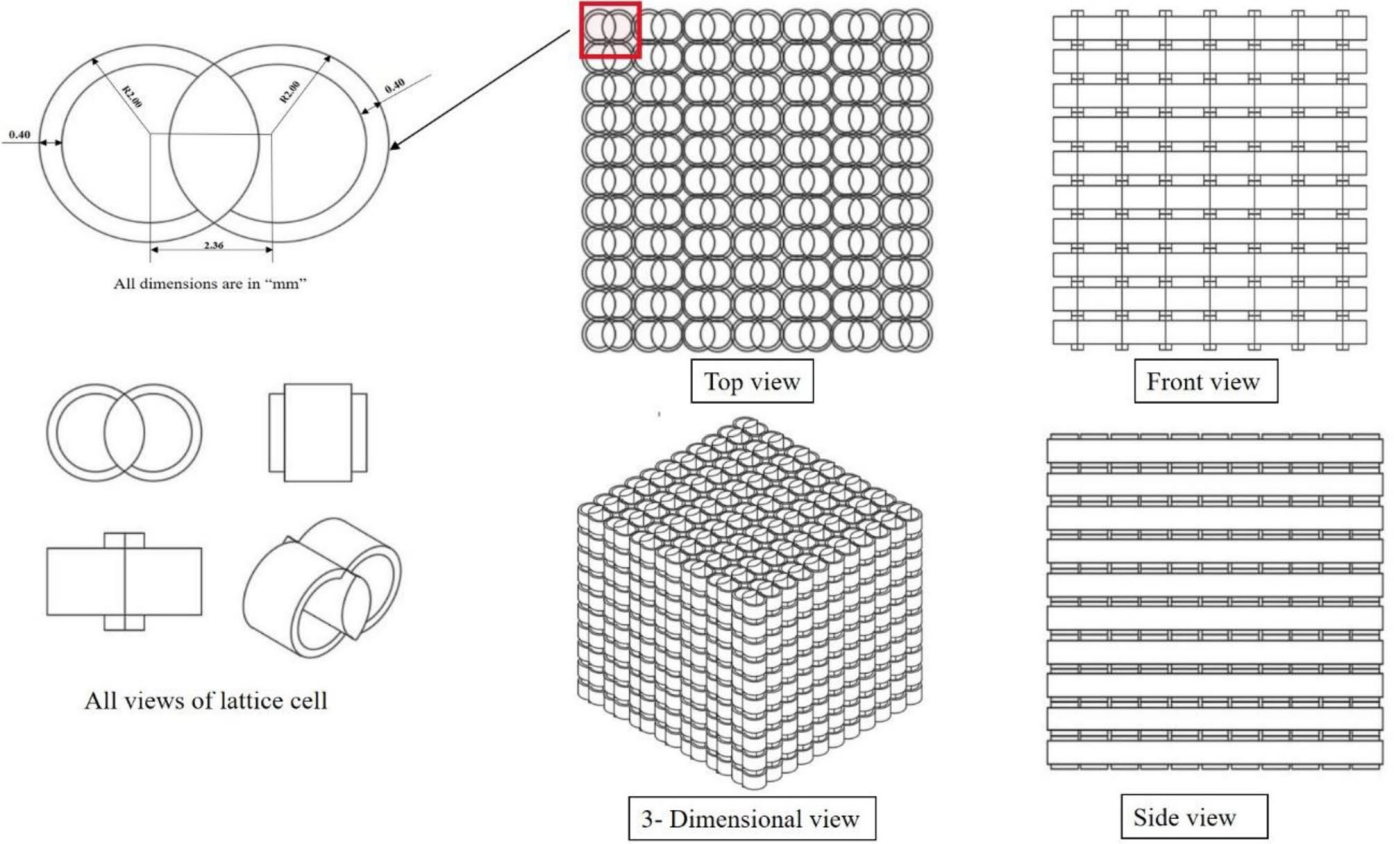
Tessellation views of the bio-inspired structures.

In the Design methodology, the circular shape is obtained from the bamboo tree structure, the overlapping phenomenon is obtained from the scales of the aquatic species, and scutes are obtained from the alligator, crocodile, and turtle species. More information regarding the overlapping phenomenon of marine species was discussed in the literature^6^. This paper shows two circular diameters with four overlapping areas and two additional circular wall thicknesses, as shown in Fig. 1. The overlapping area of the unit cell is decided by the percentage area of an individual circle with a radius ‘r’. The overlapping size in the unit cell can be controlled with the distance between the centre’s **‘d’** of the two overlapped circles. The selection approach of the parameters is considered from the inspiration of bamboo sticks. The wall thickness parameter has been permitted in the range of 0.4–0.6 mm after rigorous printing efforts. If the wall thickness is below the allowed range, the printing of lattice cells fails. In the same way, the wall thickness is above the permitted range, and constant overlap of the cells is not achieved within the given volume. The parameter circle diameter range of 4 mm to 6 mm is considered from the inspiration of bamboo stick diameters. The diameters below the considered range are unsuitable for printing as details about the lattice cells were not captured properly. In the same way, the diameters above the considered range will reduce the number of cells within the given volume, leading to a decrease in mechanical properties. A typical tessellation methodology to better understand lattice cell structures is shown in Fig. 2.

The cell is taken from the circular area of the one circular region of the radius of ‘**r**’. The overlapping site in the unit cell lattice can be controlled with the distance between the centre’s **‘d’** of the two overlapped circles. The overlapping area of the two circles is given by Eq. (1).

1 $${\mathbf{A}} = {\mathbf{2r}}^{{\mathbf{2}}} {\mathbf{cos}}^{{ - {\mathbf{1}}}} \left( {{\mathbf{d}}/\left( {{\mathbf{2r}}} \right)} \right) - {\mathbf{1}}/{\mathbf{2d}}\surd \left( {{\mathbf{4r}}^{{\mathbf{2}}} - {\mathbf{d}}^{{\mathbf{2}}} } \right)$$

where r = radius of the circle, and d = distance between the centre of the two circles.

**Figure 3:**
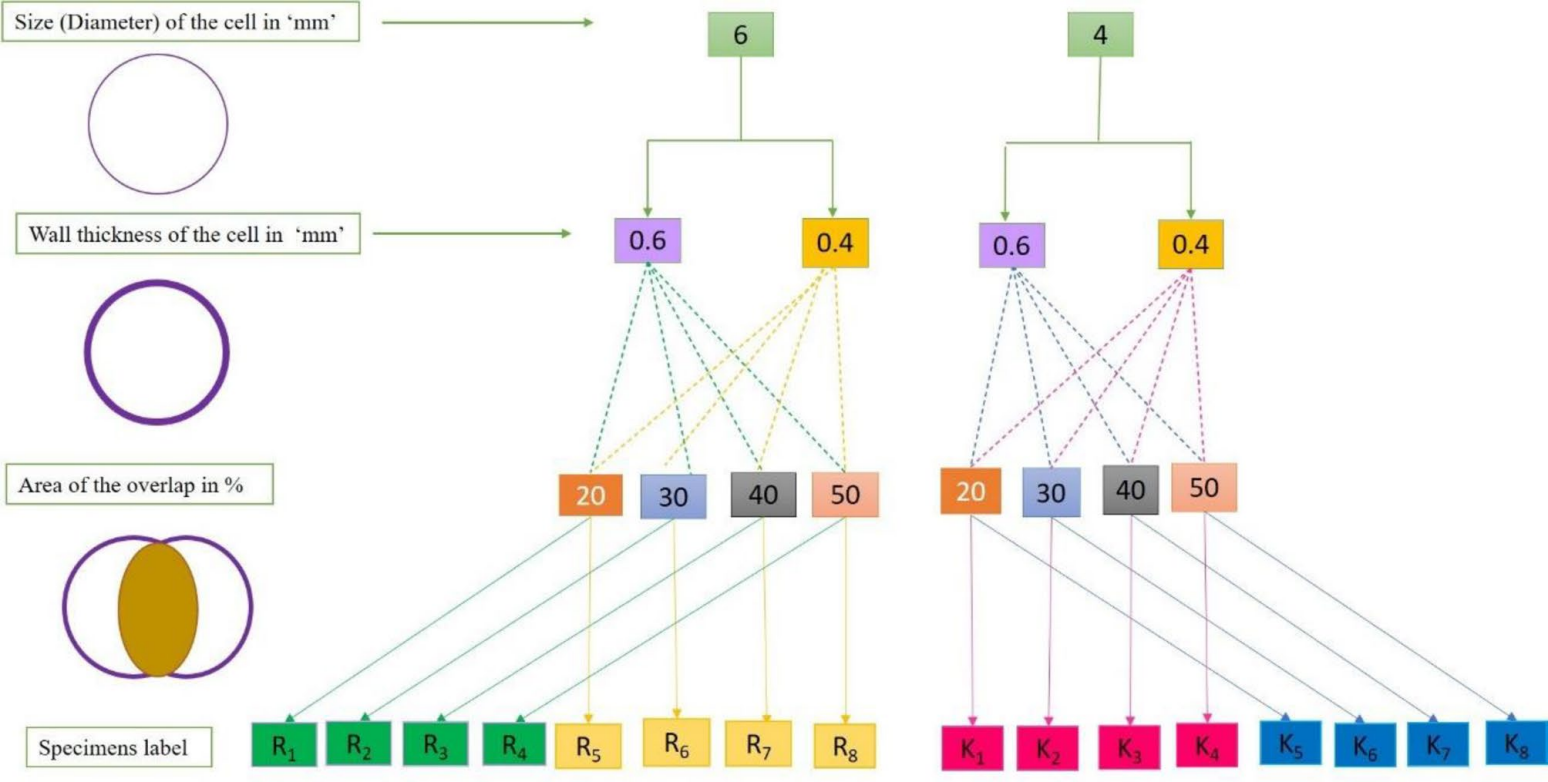
Parameters for the preparation of design and fabrication of specimens with labels.

In this paper, a total of 16 distinct types of lattice structures were developed using varied parameters, like the size of the circle, the thickness of its unit wall, the region where the circles overlap, and a limit of 6 mm for the height of the scute-like hump in each construction. All the parameters for the lattice structure are changed only within the cube size range, which also limits the size of the construction to a simple cubic of 40 × 40 × 40 mm. The size of the cell and the overlapping of circular areas are critical in the production of the unit cell count since changing the parameters in each structure may result in a different count of unit cells in each specific lattice structure. Figure 3 illustrates the labelling of each lattice structure among the 16 specimens: R1, R2… R8, and K_1_, K_2_… K_8_. The Autodesk Fusion 360 3D CAD software was used to build these novel hybrid bio-inspired lattice structures. All files were exported and saved as STL files in preparation for manufacturing using Vat polymerized 3D printing.

## Fabrication using Vat polymerization

**Figure 4:**
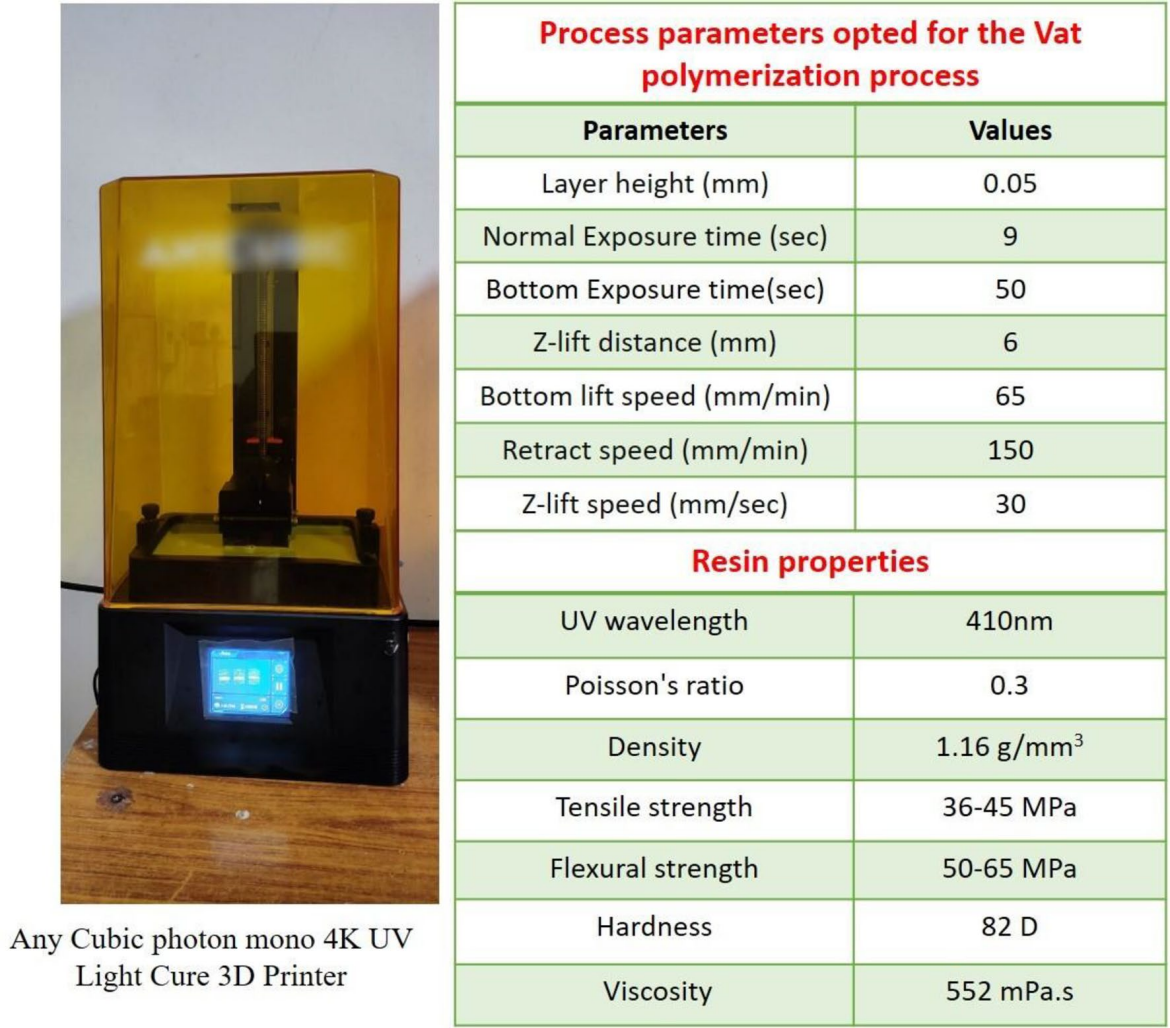
Specifications of Vat polymerization-based- three-dimensional printer and resin material.

All STL-formatted files are stored and prepared for importation into the slicing software. A compatible vat polymerization-based three-dimensional printer is employed to produce all specimens. All files are ready in the layer-to-layer format for excellent precision in dimension and structure. When producing 3D prints, all of the printing machine's parameters, including layer height, printing speed, retraction speed, UV power, bottom layer count and thickness, type of support, specimen tilt concerning the build plate, exposure duration, and lifting speed, should be accurately verified. All designed specimens are printed using any standard cubic resin utilizing any cubic photon mono x SLA 3D printer with a digital light processing approach. Figure 4 displays the parameter settings for the 3D printer, resin characteristics, and any cubic 3D printer. Any cubic photon workshop is the slicing program utilized for the slicing. Lattices began printing layer by layer after the first printing of support material that bonded to the build platform. All lattice prints were created using layers that were 50 µm thick. However, print times vary depending on the lattice design and the desired number of prints, and sometimes print failure may take more time even if one fails. Lattices were lifted off the construction platform using a metal spatula and soaked in an 80 per cent isopropyl alcohol solution for five to ten minutes. The maximum time taken for each printing set is an average of 4 h. All cleaned samples are placed in a curing chamber for 20–30 min under a UV light source or under sunlight as a free UV light source.

**Figure 5:**
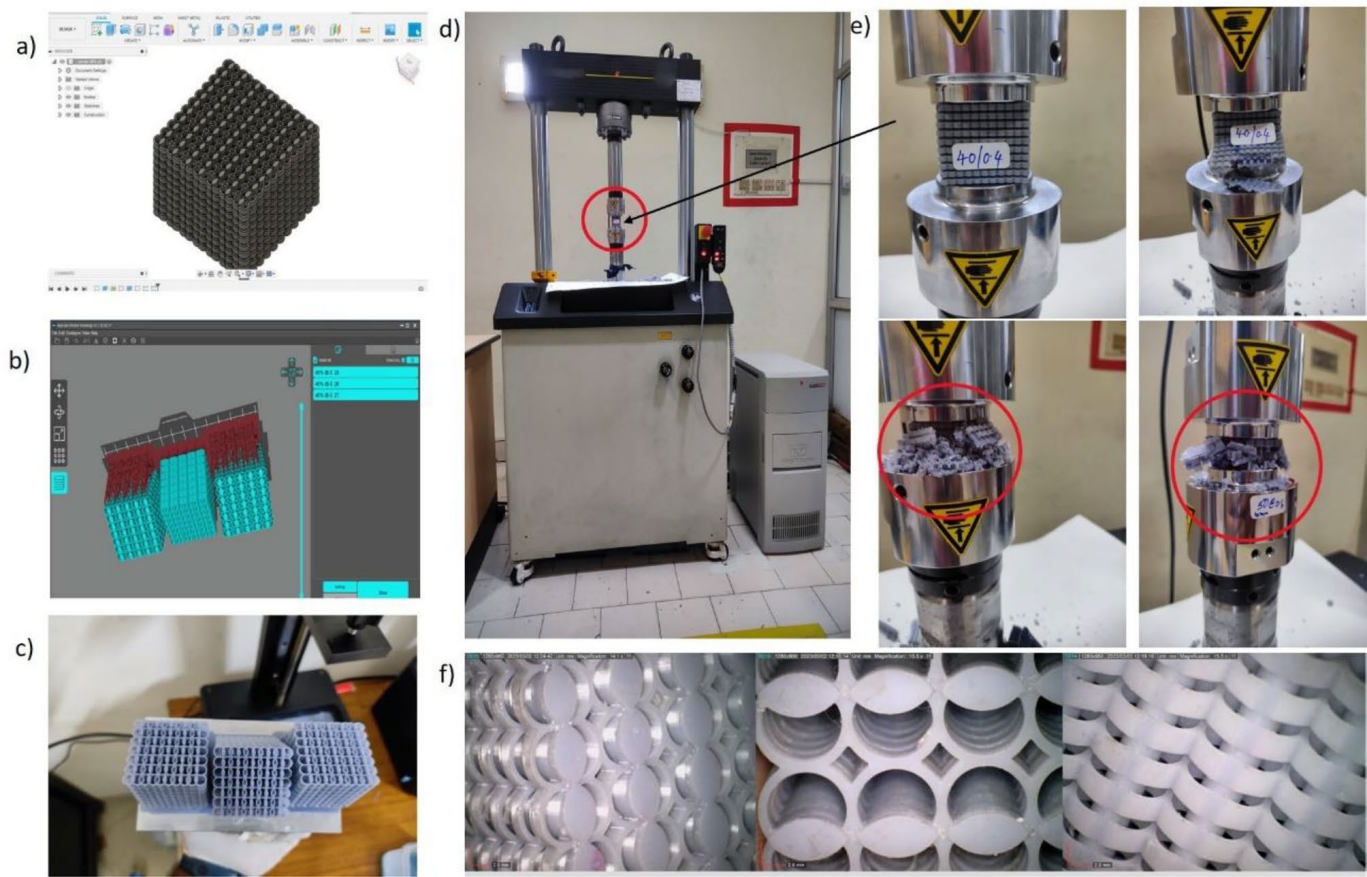
(**a**) Design and modelling of Novel hybrid Bio-inspired lattice structures, (**b**) Slicing of the 3D CAD model in slicing software, (**c**) Fabrication of specimen using a 3D printer, (**d**) Quasi-static compression testing, (**e**) Failure of 3D printed specimens under compression loading, (**f**) microscopic images of the specimens showing uniformity of material.

Using snipping tools and razor blades, extra support material was manually removed after curing, and the rough edges were then sanded to achieve the final post-processing. Every structure has essentially isotropic elastic properties with a circular cross-section. In addition, the isotropic property of the resin material with a circular cross-section (struts) is clearly discussed in the literature^25,39–42^. Microscopy images of a few samples showed that each unit cell was uniformly printed across the structure, as shown in Fig. 5f. Various defects were visible for models with damaged unit walls caused by removing support material. We utilized vernier callipers to gauge the manufacturing accuracy of the lattice and a scale to gauge the weight. This printing method may reliably manufacture components with outside size variations from the desired dimensions of less than 0.5%. Figure 5 clearly illustrates the modelling, slicing, production, and testing of the specimens.

### Experimental setup for mechanical testing

The specimens were positioned on a level platform between two compression plates during the quasi-static compression test utilizing Instron 8801 Universal Testing equipment after manufacturing and curing, as shown in Fig. 5. The force–displacement response was recorded at a 2.5 mm per minute loading rate for each sample. For each mechanical test, many specimens were printed, and the results were averaged for statistical analysis. The device can test metal and polymer materials with a consistent loading capacity of 100kN. The testing is done following ASTM standard D1621. During the compression, the extensometer measured the deformation curves and stress–strain values, which is connected to the experimental system. The tests are run a minimum of five times with the same displacement to confirm the accuracy and repeatability of the results.

All structural samples have reached 60% to 70% densification after the top plate has moved 26–28 mm during the compression testing, as shown in Fig. 5e, which is observed in the red marking during the failure of the specimens and more data regarding the densification of the specimens can be seen in the previous research^42^. Blue Hill software, which Instron UTM supports, was used to record the stress–strain curves acquired from the apparatus. The given set of all specimens underwent testing, as illustrated in Fig. 5. The graphs are constructed using all the experimental data collected from the Blue-hill software.

## Results and discussion

**Figure 6:**
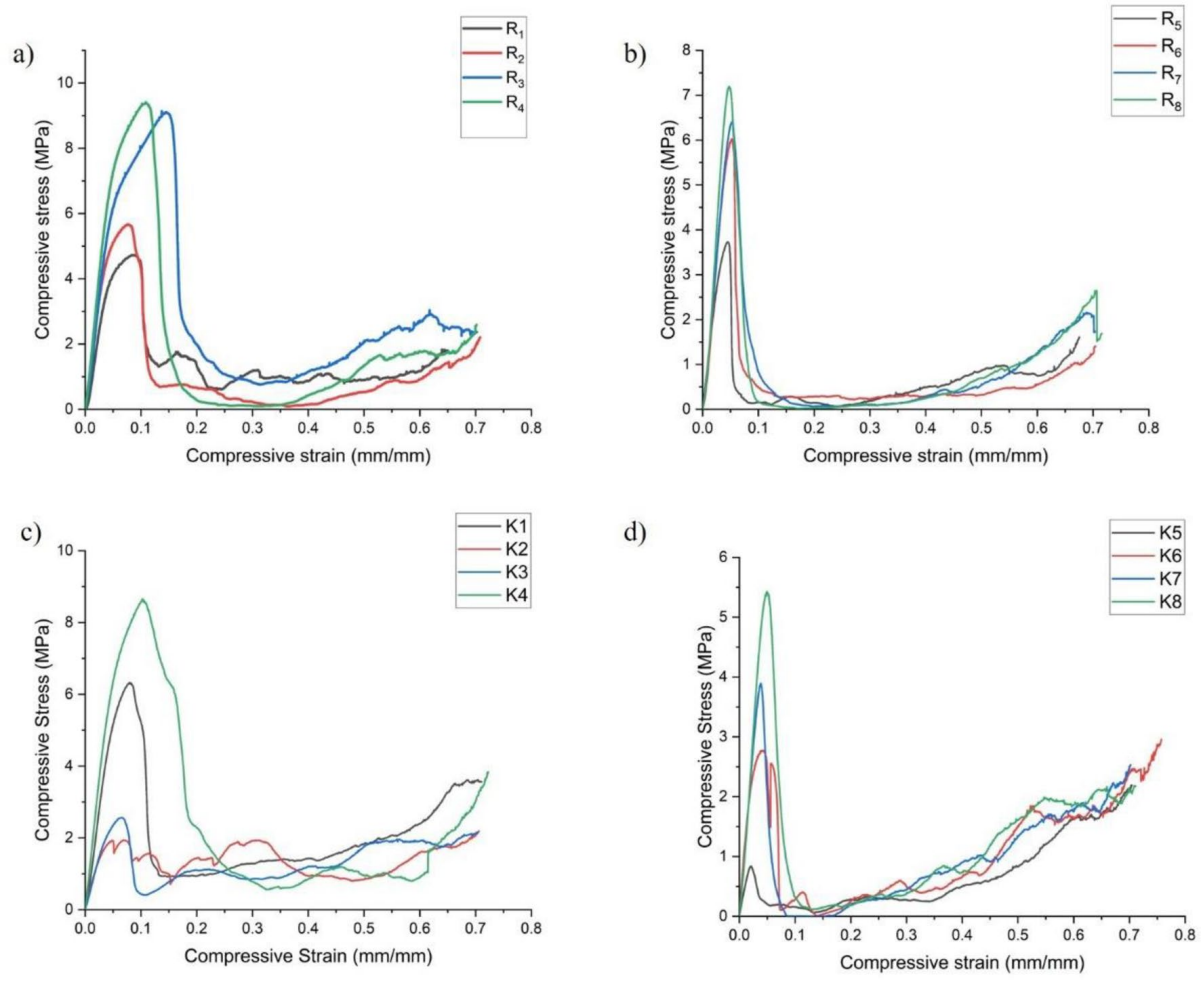
Graphical curves showing the stress–strain variation of (**a**) Specimens R_1_–R_4_, (**b**) Specimens R_5_–R_8_, (**c**) Specimens K_1_–K_4_, (**d**) Specimens K_5_–K_8_.

In this paper, the properties of three-dimensional printed lattice structures were examined, as well as their prospective usage for energy absorption applications. The TEA of all the designed bio-inspired novel lattice structures has been measured from curves traced between strain and stress values shown in Fig. 6. Further, Total energy absorption (TEA) values were calculated using Eq. (2).

2 $${\mathbf{TEA}} = \smallint {\mathbf{F}}_{{{\mathbf{av}}}} {\mathbf{d}}_{{\mathbf{s}}} \equiv \, {\mathbf{F}}_{{{\mathbf{av}}}} *\left[ {{\mathbf{d}}_{{\mathbf{f}}} - {\mathbf{d}}_{{\mathbf{i}}} } \right]$$

The F_av_ is the mean crushing load, d_f_ is the final crushing distance, and d_i_ is the initial crushing distance.

**Figure 7:**
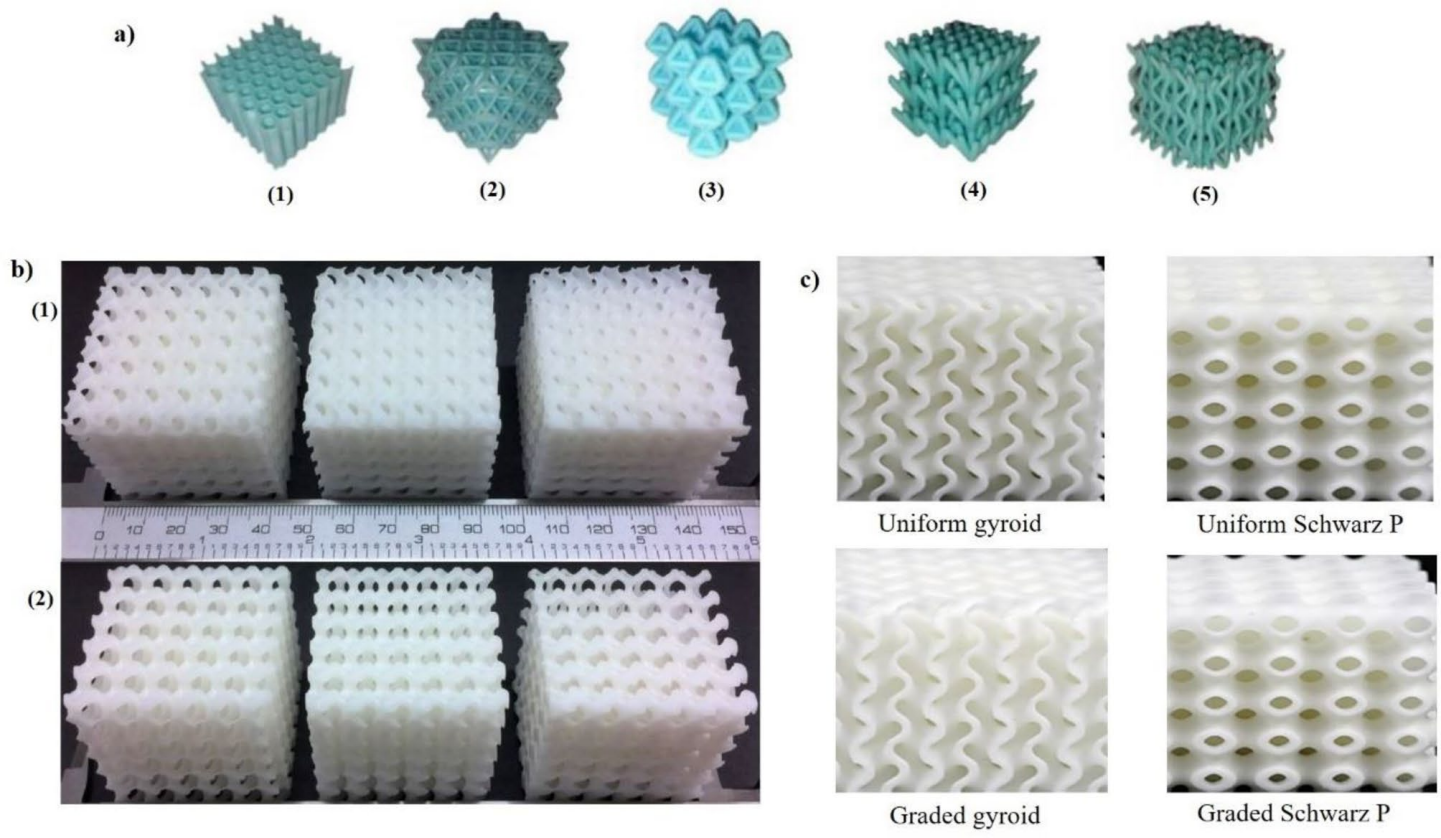
Three-dimensional printed samples of (**a**) Typical strut-based lattices (1) Honeycomb (2) Triple Merged tessellated lattices (TMTL) (3) Doubly merged tessellated lattices (DMTL), (4) single dimensional flower lattice (1-DF), (5) two-dimensional flower(2-DF)^25^, (**b**) (1) sheet based gyroid structures, (2) strut based gyroid structures^43^, (**c**) uniform and graded gyroid and Schwarz p structures^44^.

The energy absorption behavior of the proposed lattice structures is compared with the typical bio-inspired strut-based lattice structures investigated in the recent literature^25^ as shown in Fig. 7a. In addition, triply periodic minimal surface (TPMS) structures like strut-based and sheet-based gyroid structures discussed in the literature^43^ as shown in Fig. 7b and the typical TPMS with large surface areas and zero mean curvature (i.e., uniform gyroid and Schwarz-p, graded gyroid and Schwarz-p) discussed in the research^44^ as shown in Fig. 7c. In proposed lattice structures, the energy absorption of specimen K4 has resulted in more than 1.8 kJ/Kg whereas the structures discussed in the literature^25,43,44^ recorded less than these values at a lattice densification of 60% from the quasi-static compression test results.

### Relative density of the lattice structures

**Figure 8:**
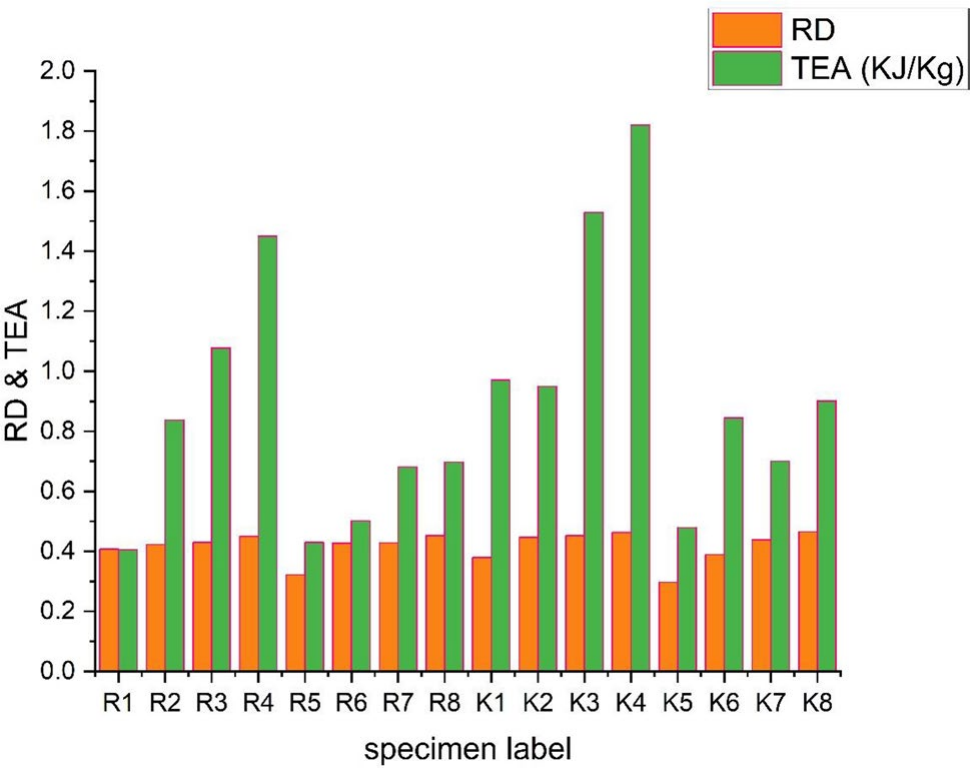
Graph showing the variation of Relative density (RD) and TEA values of the lattice structures.

The Relative Density (RD) is one of the influencing parameters to define the properties of a lattice structure. The RD of a structure can be calculated based on the weight or volume of the structure. In this work, RD is calculated on the basis of the volume of each lattice structure and is compared with the solid cubic of 40 mm size where the total volume of a solid cube is 64000mm^3^. The values of RD of better-performed structures with the variation of the energy absorption concerning the relative density are detailed in the graph, as shown in Fig. 8. Equation (3) gives the formula for finding the structures' RD.

3 $$\mathrm{Relative\,Density}=\frac{Density\,of\,the\,lattice }{Density\,of\,the\,solid}$$

From the graph shown in the figure, the RD values of all the lattice structures show almost in the approximate range, and TEA values associated with each sample were also clearly visible where K_4_ and K_3_ are given a better performance. The mechanical properties depended on the relative density values of the current research and were compared with the recent research work developed in the literature^22,23,39–41^. The performance results of this study hold good from the perspective of lattice structures.

The data obtained from the above process is summarised and applied to predict the output TEA of structures with the known inputs using one of the best machine learning techniques, the Artificial Neural Networks tool. In this study, we are applying the Levenberg–Marquardt Algorithm (ANN-LM) training algorithm. To get better results for running this type of algorithm K-fold Cross-Validation technique is used.

### Levenberg–Marquardt algorithm (ANN-LM)

**Figure 9:**
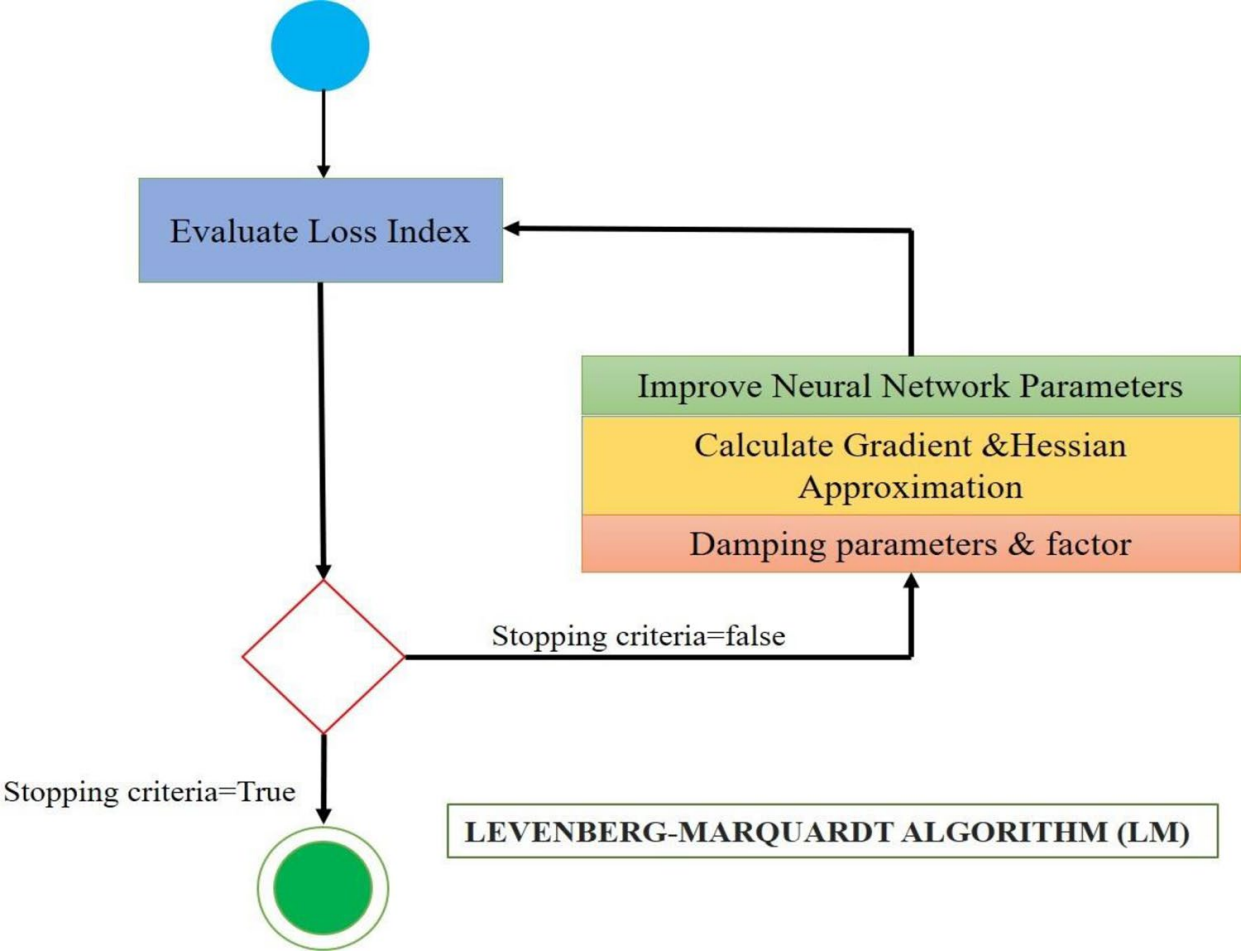
Flow chart for Levenberg–Marquardt Algorithm (ANN-LM).

The damped least squares method is technically named Levenberg–Marquardt Algorithm (ANN-LM), which resolves the damped least squares approach. A detailed flowchart for the ANN-LM algorithm is shown in Fig. 9.

Rather than calculating the precise Hessian matrix, this approach computes the matrix using the Jacobian matrix and gradient vector. An expression for the loss function is the sum of squared errors as

$$f=\sum_{i=1}^{k}{e}_{i}^{2}$$

where ‘**k’** is the number of training samples, and **‘e’** is the vector of all error terms.

The loss function's Jacobian matrix is described as follows:

$${\mathbf{J}}_{i,j}=\frac{\partial {e}_{i}}{\partial {\mathbf{w}}_{j}},$$

where i = 1…, a and j = 1.., b and an is the Jacobian matrix, b is the count of parameters present in the neural network, and a is the number of cases in the dataset. The Jacobian matrix has a size of [a, b]. The loss function's gradient vector is computed as

$$\nabla f=2{\mathbf{J}}^{T}\cdot \mathbf{e}$$

Approximately, the Hessian matrix is calculated by

$$\mathbf{H}f\approx 2{\mathbf{J}}^{T}\cdot \mathbf{J}+\beta \mathbf{I}$$

H is the identity matrix, and I is the damping factor, assures the Hessian's positive sign. The first stage is the selection of a significant parameter.

Then, it will be increased by a certain amount if there is a mistake in any iteration. On the other hand, if the loss goes down, it will also go down, bringing the Levenberg–Marquardt algorithm closer to the Newtonian approach. The Levenberg–Marquardt algorithm is used to enhance the parameters, and it is defined as

$${\mathbf{w}}^{(i+1)}={\mathbf{w}}^{(i)}-({\mathbf{J}}^{(i)T}\cdot {\mathbf{J}}^{(i)}+{\beta }^{(i)}\mathbf{I}{)}^{-1}\cdot (2{\mathbf{J}}^{(i)T}\cdot {\mathbf{e}}^{(i)}),$$

for i = 0, 1.

For the first updates to have tiny steps in the gradient descent direction, the parameter $\beta$ is initialized to be significant. If any iteration fails, then $\beta$ is raised by a certain amount. Otherwise, $\beta$ is lowered as the loss reduces, and the Levenberg–Marquardt algorithm gets closer to the Newton technique. Usually, this technique speeds up convergence to the minimum. Figure 9 shows the state diagram for the Levenberg–Marquardt algorithm's training of a neural network. Calculating the loss, gradient, and Hessian approximation is the initial step. The damping parameter is then modified to minimise the loss throughout each repetition. A technique made specifically for functions of the type sum-of-squared error is the Levenberg–Marquardt algorithm. This makes it incredibly quick to train neural networks based on mistakes of that nature.

### K-fold cross-validation

**Figure 10:**
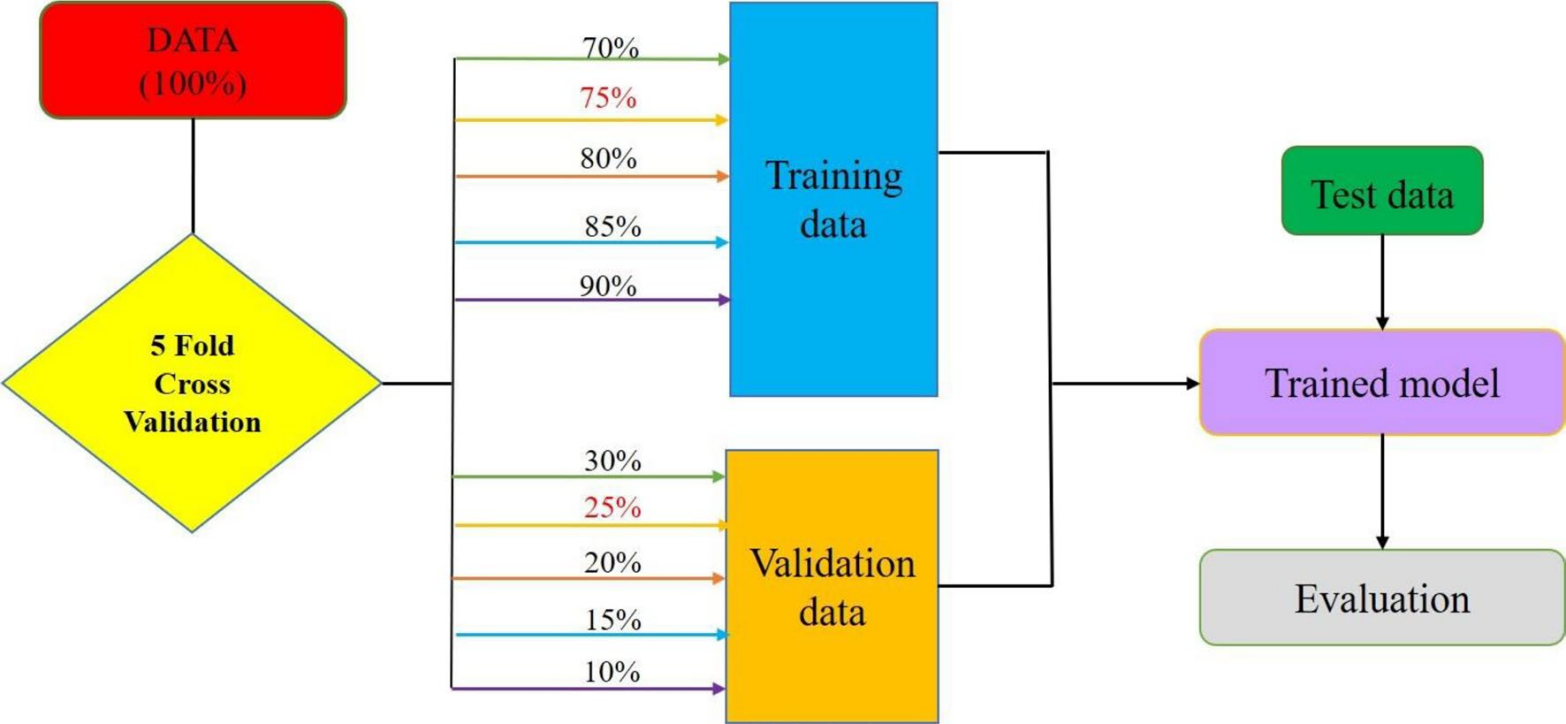
Five-fold cross-validation for data training.

Cross-validation is a machine-learning assessment technique used to determine how effectively your machine-learning model can forecast the result of data that has not yet been observed. It is a popular option since it is simple to understand, performs well for a small data sample, and provides a less biased evaluation. In this paper, fivefold cross-validation is attempted by choosing the k value as 5. The dataset is divided into 5 folds during cross-validation, which assesses the model’s performance when faced with new data. The total number of groups into which the data sample is divided is 5. Every Data Scientist should utilise k -fold cross-validation or at least be highly skilled. It enables you to use your data more effectively and gives researchers, data scientists, and engineers working in machine learning a better knowledge of how well the algorithm performs. The proposed fivefold cross-validation applied in the ANN is shown in Fig. 10.

**Figure 11:**
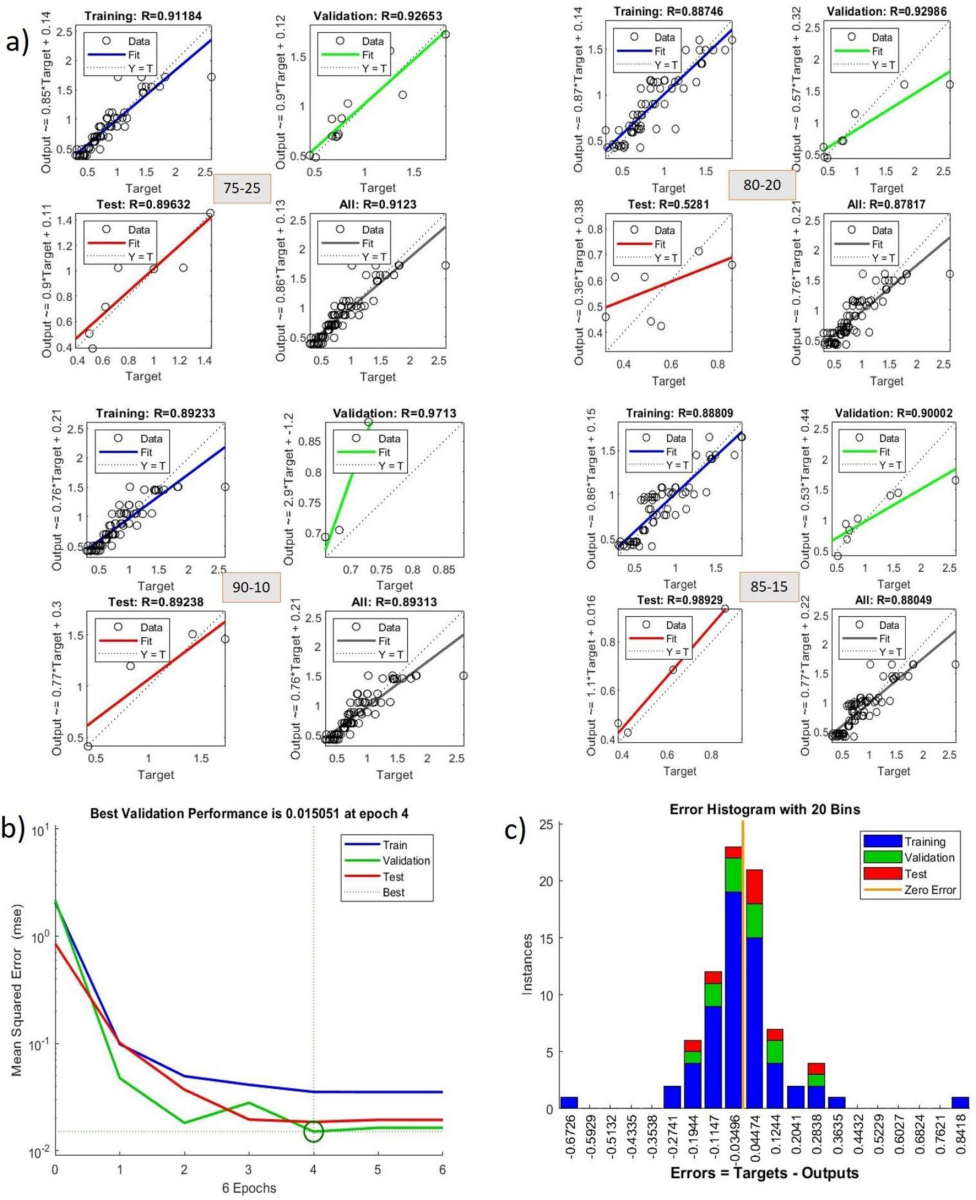
(**a**) Regression values of different data sets (**b**) performance curve traced for set 75–25 (**c**) Histogram error shown for set 75–25.

The figure shows that 5 sets of test data are applied to train the algorithm for the best results to predict the output results. Training of the available data using 5 -fold cross validation is carried out using Levenberg–Marquardt Algorithm (ANN-LM) in MATLAB software. Overall, among 5 training data test results, the set with 75–25 shows the best R values with better training performance with the minor histogram error, as shown in Fig. 11.

**Figure 12:**
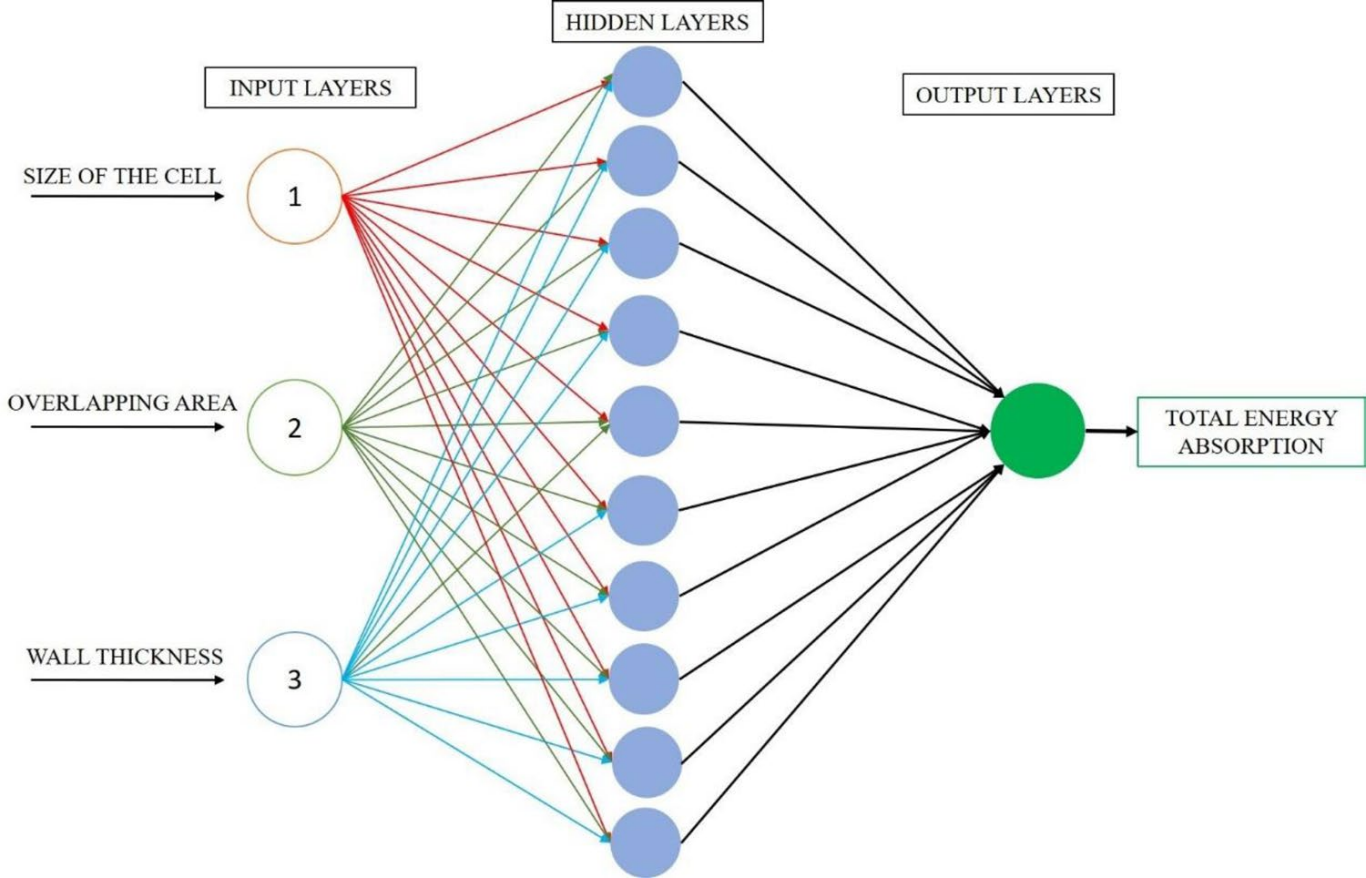
ANN showing Input layers, Hidden layers, and output layers.

An ANN model must include output and input components, with the former being controlled by the latter. The ANN principle is a network of interconnected neurons carrying weights. This indicates that to get the model's response, the weight must be multiplied by the total number of signals in the network. The input, hidden, and output layers are the three layers of a primary ANN network. The input and output layers are established before data training, whereas trial and error discover the hidden layer throughout training and testing. The training process with input layers, hidden layers, and output layers of the training algorithm is clearly shown in Fig. 12. The study has demonstrated that, despite the presence of mistakes or missing data in the data being processed, the ANN approach can foretell substantial reactions. The three stages of the ANN methodology are model performance evaluation, training, and learning. To ensure the predictability of the output variables, the network's biases, and weights (unsupervised or supervised) are changed throughout the training phase. While the supervised training method uses previously completed experimental data to create a model, where the real-world output and input data are not employed in unsupervised training. The network may react to information during testing without completely altering its architecture. At the end stage of each development, the optimal ANN model may be found through a process of tries and mistakes. It was determined that it was feasible to keep creating a significant count of networks at some time throughout the learning process. At that point, the process could be halted and evaluated at different points during the learning process.

Re-examine the network with various random weights and keep doing so until you obtain the desired outcomes. The most crucial parameters to consider while choosing the optimum neural network model are the result coefficient (R^2^) and mean square error (MSE). The effectiveness of the ANN model was evaluated using the MATLAB program. The model was constructed using the straining and recall methods and the error backpropagation strategy.

**Figure 13:**
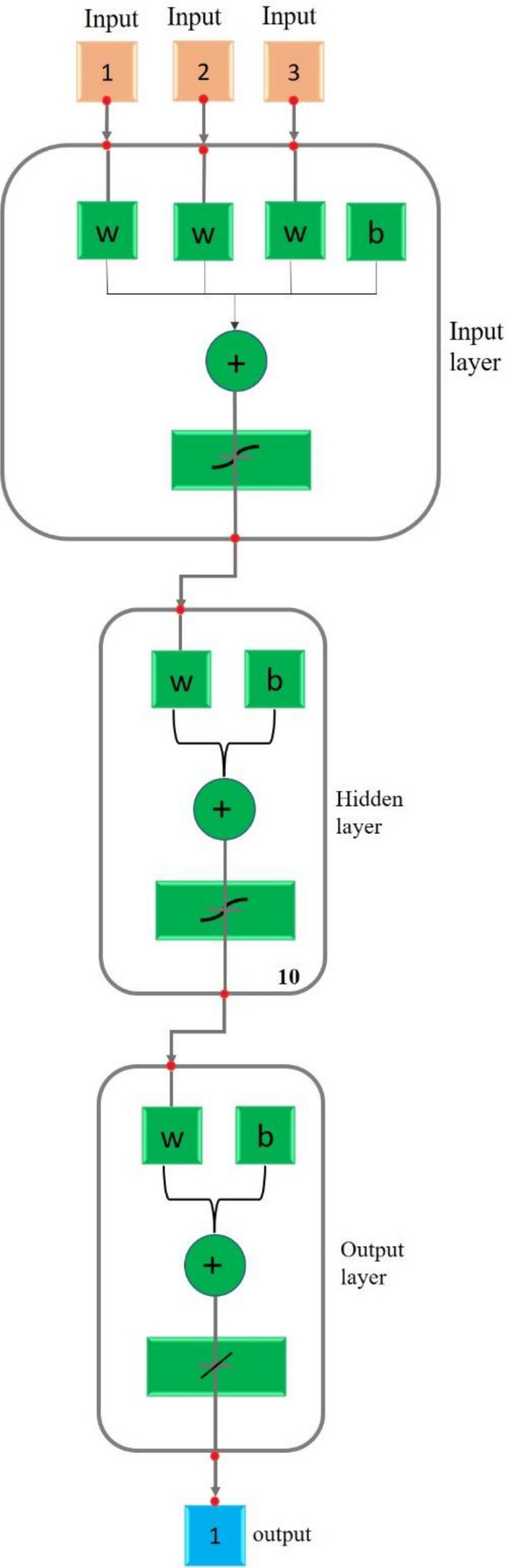
ANN model developed in MATLAB software using LM algorithm.

The data was trained using an LM multilayer feed-forward back-propagation model and is readily available in the MATLAB computing environment. The information for this study’s conclusions was compiled from 80 samples. Twenty-five per cent of the data was used in each testing and validation phase, and seventy-five per cent in the learning phase. The data was automatically normalised rather than spitted manually using the maximum data values, as was performed in MATLAB. The process was repeated several times till the optimal model that satisfied the MSE and R^2^ requirements was identified. When a model’s MSE is minimal, and its R^2^ is close to 1, It shows a perfect correlation between the expected and observed data. It is generally accepted that the more effective an ANN model is in predicting the future behaviour of a system, the better. The neural network model obtained from the training algorithm is shown in Fig. 13.

The predicted and actual testing findings on the energy absorption capacity of the lattice structures are very comparable, as evidenced by the performance of the selected network design. The chosen model may offer input and output data for the evaluated concrete while maintaining high accuracy in its predictions. A less matching per cent error further supports the model’s utility for the projected strength, which shows that the forecast produced by this model is statistically accurate. Due to the close closeness of the datasets, this result demonstrates that the model’s effectiveness may be depended upon in absolute terms (actual and predicted). This shows that the chosen model can respond to the system correctly and with high confidence in its performance. Final trained data with 3 input layers,10 hidden layers and one output layer generated in the software for training, testing and validation is shown in Fig. 13.

**Figure 14:**
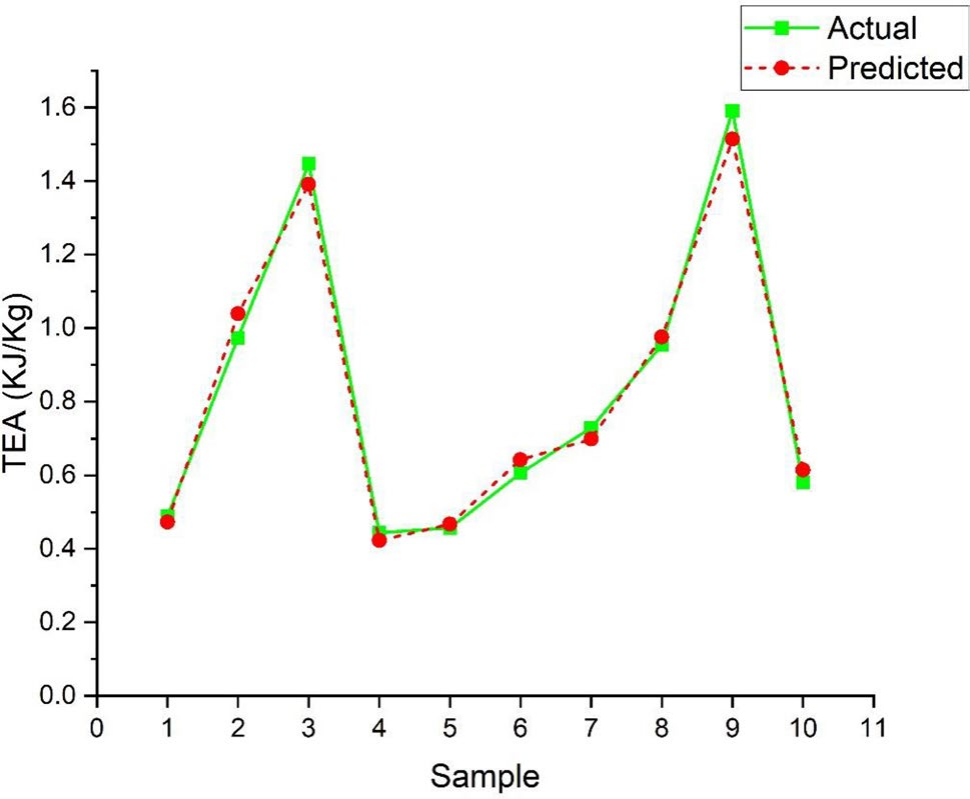
Shows the variation of actual values with predicted values for the ANN-LM Algorithm.

A graph was plotted between the predicted and actual values randomly chosen from the 80 samples calculated from the TEA Eq. (2) for a transparent verification for data training. Negligible errors were observed from the real and predicted values from the available data set of input and output values, as shown in Fig. 14.

### Prediction and experimental validation

For the trained algorithm, separate raw data of input values have been assigned for predicting TEA values as output, as shown in Table 1.

**Table 1:** New set of input data with prediction values.

Size (Diameter) of the cell	Wall thickness of the cell in ‘mm’	Area of the overlap in %	ANN-LM Predicted Values
4	0.4	35	0.9538
4	0.6	25	1.0185
4	0.6	45	0.6915
5	0.4	40	0.8022
5	0.6	25	0.46995
5	0.6	30	0.6865
6	0.4	45	1.7813
6	0.6	25	0.9585
6	0.6	35	0.651183

**Figure 15:**
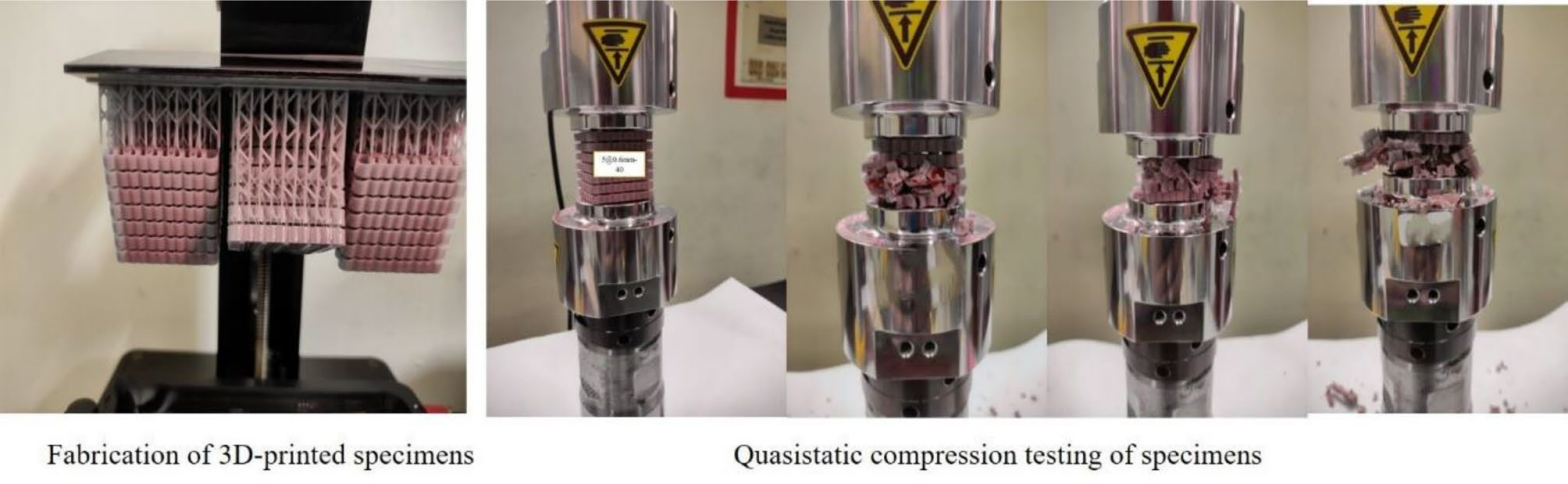
Fabrication and testing of the specimens to compare with predicted values.

With the same input raw data, the structures are again modelled, printed using the Vat polymerization technique, and tested under quasi-static compression loading, as shown in Fig. 15. The results are obtained from the UTM equipment, and energy absorption capacity values of all tested specimens are calculated and compared with the predicted values.

**Figure 16:**
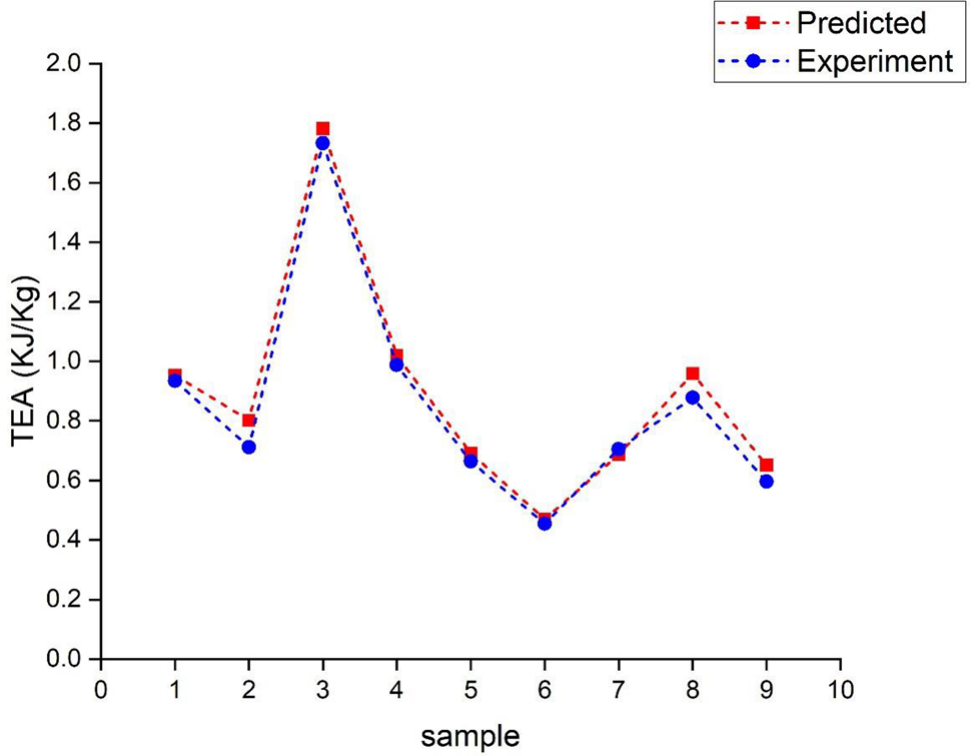
Graph showing the variation of predicted and experimental test values.

The graph in Fig. 14 shows the variation of actual values with the prediction values after training of the available data set is completed. Actual and predicted values are verified to know the better accuracy of the trained algorithm called the Levenberg–Marquardt Algorithm (ANN-LM) by applying k-fold cross-validation where the k value is set for five. This graph is plotted by taking random samples of the available data. It was fascinating that the predicted and actual values showed a low error in many sets, less than five percent. So, it was a heavy boost to the study that the algorithm is well-trained for testing and validation data. In a deep, it was noticed that an error in the values of samples 2, 3, and 9 was observed from the graph that is considered negligible. From the satisfactory results obtained from the above case, the algorithm is again tested for the new set of values, as shown in Table 1. These input values are dumped into the trained algorithm, and output results are noticed by repeating several runs for better accuracy of the output values. The predicted values obtained from the repeated runs were averaged to one value for all runs of input data sets, and corresponding predicted values are shown in Fig. 16.

A graph was plotted between the predicted values and experimental values of the new set of data shown in Fig. 16. From the graph, it was observed and very clear that there is a good convergence happening among the prediction and tested values. The trained algorithm with test and validation data was run prominently with the new input parameters and appropriate output results.in the depth scene of the graph, almost all samples reached the prediction values of TEA. Samples 2 and 8 only showed the visible error in the output values. These errors can be reduced to zero tolerance by handling the fabrication and experimental testing of the specimens in real time. The input parameters are applied in this research limited to small values only because the size of the unit lattice cell and the count of unit lattice cells are significant considerations for the property of energy absorption capacity of the cellular lattice structures. Maintaining the cell's optimum size and unit cell number is vital in this connection while designing lattice structures for all structural applications.

## Conclusion

This research study created and modelled a novel hybrid lattice cellular structure in design software. All designs were sliced into layers as STL files by slicing software, and the designed specimens were fabricated using the Vat polymerization technique. Then, after specimens are tested under quasi-static compression via the Instron UTM machine, the results obtained from this test are used to calculate the energy absorption values of all individual specimens. The significant outlines which are drawn from this research are summarized as follows:

- Artificial neural networks with as Levenberg–Marquardt Algorithm (ANN-LM) are one of the best machine learning tools used to train with the available data, including input and output values with varied training sets.
- The k-fold cross-validation technique is implemented in this paper with a k value of 5. The best training set was recorded with 75% training data and 25% test data.
- Algorithm was trained with several runs with available data and obtained predicted values were compared with the actual data for better accuracy of the trained algorithm.
- New raw input data was given to the ANN-LM algorithm, and output-predicted results were noted and compared with the experimental results. It was fascinating that the predicted values in both cases are good with the actual data and experimental results, as shown in the graphs.
- Additionally, the ANN model might be valuable for analyzing the error between predicted and real-time results. ANN-LM is an intriguing numerical tool that might significantly reduce the need for expensive and time-consuming experimental and simulation techniques. Even though several training procedures and epochs have been investigated, this work only uses one ANN design.
- Even though the best and most impressive accuracy in prediction is achieved, it is fascinating to conduct additional research on the number of neurons and hidden layers, potentially improving the ANN-LM model performance or cutting down on computing time by reducing the number of neurons in the hidden layer.
- To improve prediction accuracy, future studies will consider the manufacturing flaws of the structures and the processing factors of the innovative lattice structures in various additive fabrication procedures. Lattice structure failure mode prediction research will be interesting as well.

## Supplementary Information

Supplementary Information.

## Data Availability

The datasets used and analyzed during the current study are available from the corresponding author upon reasonable request.
