# Supplementary material for: Prediction and experimental validation approach to improve performance of novel hybrid bio-inspired 3D printed lattice structures using artificial neural networks
Source: Sci Rep. 2023 May 12;13:7763. doi: 10.1038/s41598-023-33935-0 (PMC10182031; doi:10.1038/s41598-023-33935-0)
Supplement: Supplementary file 1 — Supplementary Information. [file 41598_2023_33935_MOESM1_ESM.pdf]

Data set used to train ANN-LM

SIZE OF CELL	WALL THICKNESS	AREA OF OVERLAP	TEA in KJ/Kg
6	0.6	20	0.489138371
6	0.6	20	0.300000578
6	0.6	20	0.426731051
6	0.6	20	0.457934711
6	0.6	20	0.363365814
6	0.6	30	0.729076118
6	0.6	30	0.775528856
6	0.6	30	1.006065026
6	0.6	30	0.867570572
6	0.6	30	0.890796941
6	0.6	40	0.973077344
6	0.6	40	1.386757768
6	0.6	40	0.872994889
6	0.6	40	0.923036116
6	0.6	40	1.129876328
6	0.6	50	1.458288867
6	0.6	50	1.44660066
6	0.6	50	1.443880473
6	0.6	50	1.45108467
6	0.6	50	1.445240566
6	0.4	20	0.52040223
6	0.4	20	0.443997105
6	0.4	20	0.323830665
6	0.4	20	0.422116447
6	0.4	20	0.383913885
6	0.4	30	0.51566989
6	0.4	30	0.533243416
6	0.4	30	0.456906694
6	0.4	30	0.486288292
6	0.4	30	0.495075055
6	0.4	40	0.631085357
6	0.4	40	0.606422681
6	0.4	40	0.604394403
6	0.4	40	0.61773988

6	0.4	40	0.605408542
6	0.4	50	0.729092685
6	0.4	50	0.628477492
6	0.4	50	0.735439824
6	0.4	50	0.732266254
6	0.4	50	0.681958658
4	0.6	20	1.234065227
4	0.6	20	0.721933205
4	0.6	20	0.953431568
4	0.6	20	1.093748398
4	0.6	20	0.837682387
4	0.6	30	0.831011174
4	0.6	30	0.843738773
4	0.6	30	1.173510052
4	0.6	30	1.002260613
4	0.6	30	1.008624413
4	0.6	40	1.591155071
4	0.6	40	1.73420173
4	0.6	40	1.261103199
4	0.6	40	1.426129135
4	0.6	40	1.497652464
4	0.6	50	1.826804611
4	0.6	50	2.613417532
4	0.6	50	1.019387856
4	0.6	50	1.423096234
4	0.6	50	1.816402694
4	0.4	20	0.403372376
4	0.4	20	0.319333995
4	0.4	20	0.714743629
4	0.4	20	0.559058002
4	0.4	20	0.517038812
4	0.4	30	0.720422904
4	0.4	30	1.137547751
4	0.4	30	0.677839345
4	0.4	30	0.699131124
4	0.4	30	0.907693548
4	0.4	40	0.659348703

4	0.4	40	0.859973085
4	0.4	40	0.580678212
4	0.4	40	0.620013458
4	0.4	40	0.720325648
4	0.4	50	0.771954496
4	0.4	50	0.62165644
4	0.4	50	0.719031254
4	0.4	50	0.745492875
4	0.4	50	0.670343847
